# Statistical Analysis of Noise Propagation Effect for Mixed RF/FSO AF Relaying Application in Wireless Sensor Networks

**DOI:** 10.3390/s20040979

**Published:** 2020-02-12

**Authors:** Dae-Kyo Jeong, Cheol-Sun Park, Dongwoo Kim

**Affiliations:** 1 Department of Electronics and Communication Engineering, Hanyang University, Ansan 15588, Korea; daekyo12@hanyang.ac.kr; 2 Agency for Defense Development, Daejeon 34186, Korea; csun1402@add.re.kr; 3 Division of Electrical Engineering, Hanyang University, Ansan 15588, Korea

**Keywords:** wireless sensor networks, mixed RF/FSO, AF relaying, power-constrained amplifying gain, noise propagation effect, exact CDF expression, performance evaluation

## Abstract

In this paper, we investigate the so-called noise propagation effect in a mixed radio-frequency/ free-space optical (RF/FSO) amplifying-and-forwarding (AF) relaying system that is applied for data transmission in wireless sensor networks. The noise propagation could be essentially severe when battery-charged sensor nodes have very limited transmit power. We provide an exact expression on the cumulative distribution function (CDF) of end-to-end signal-to-noise power ratio (SNR) for a dual-hop mixed RF/FSO AF relaying system. We assume a tightly power-constrained amplifying gain at the relay, which has been usually ignored in existing performance studies for the mixed RF/FSO AF system. It however should be considered to properly evaluate the noise propagation effect especially if the relaying power is not infinite or the sensor has a poor budget in transmit power. We apply the derived exact CDF to evaluate the system performances such as outage probability, average bit-error rate, and ergodic capacity. Numerical investigation is used to justify that the proposed analysis is exactly matched with the simulation and shows that the performance gap caused by the inclusion of the noise propagation effect is significant (about 2–12%) especially when the SNR per hop is in the medium- or the low-SNR ranges (i.e., at 10–20 dB).

## 1. Introduction

Increasing demand for deploying wireless sensor nodes usually raises battery and spectrum issues. An idea of reducing battery consumption in sending the sensed data is to put a relay node, which is easily charged, between the sensors and the data sink. Minimizing the transmit power at the sensors with an intermediate relay has been investigated in [1,2]. The relay could be regarded as a gateway node that provides an interface between a group of sensor nodes and the data center network. Since wireless sensors normally exchange the data through RF channels, to mitigate a spectrum burden between the relay and the data sink, free-space optical (FSO) communication is considered in this paper. FSO used instead of RF also can enhance transmission security by letting eavesdroppers hard to access the communication link.

FSO communication is already known as a cost-effective way of constructing a high-speed data tunnel between radio-frequency (RF) access network and optical fiber-based backbone network [3]. Mixed RF-FSO systems have attracted a growing interest in a dual-hop configuration to support both RF-to-FSO and FSO-to-RF relaying. As a relaying technique for the mixed RF-FSO systems, both decoding-and-forward (DF) and amplifying-and-forward (AF) relaying have been widely considered under the assumption of either intensity-modulation/direct-detection (IM/DD) or heterodyne detection (HD) in the FSO reception side. HD is known to be more complicated than IM/DD while HD offers superior performance [4].

As RF link is affected by severe fading, FSO link experiences a similar fading phenomenon, so-called atmospheric turbulence that causes fluctuations in the refractive index [5]. Furthermore, FSO link also suffers from the pointing error that refers to the misalignment between the transmitter and the receiver due to dynamic wind loads or weak earthquakes [6]. In the evaluation of the mixed RF-FSO dual-hop systems in previous studies, RF link was represented by various models including Rayleigh, Rician, κ-μ, Nakagami-*m*, generalized Nakagami-*m*, generalized-*K* and Extended Generalized-*K* (EGK) fading. On the other hand, FSO link is assumed to suffer from Mâlaga-*M*, exponentiated-Weibull, Gamma-Gamma with pointing errors, and Double Generalized Gamma (DGG) (see [7] and the references therein).

In the dual-hop AF systems, the signal from the source received at the relaying node after suffering from a fading channel is amplified with gain *G*. Since the maximum output power at the relay is usually limited, the amplifying gain should be bounded by

(1) $$\begin{array}{c} G\leq \sqrt{\frac{P_{r}}{P_{s}{\left|h_{1}\right|}^{2}+\sigma }}\overset{\mathrm{def}}{=}G^{U}, \end{array}$$

where $P_{s}$ and $P_{r}$ are transmitted power at the source and the maximum available power at the relay, respectively, $|h_{1}{|}^{2}$ is the channel power of the first hop and σ is the noise power level [8,9]. When the maximal gain $G^{U}$ is used, the end-to-end signal-to-noise power ratio (SNR, $\gamma_{e}$) of the system is represented by

(2) $$\begin{array}{c} \gamma_{e}=\frac{\gamma_{1}\gamma_{2}}{\gamma_{1}+\gamma_{2}+1}, \end{array}$$

where $\gamma_{1}$ and $\gamma_{2}$ are the SNR of the first and the second hop, respectively. For mixed RF/FSO systems, many works have been done to provide statistical analysis of $\gamma_{e}$ that mainly determines the system performance including outage probability (OP), average bit-error rate (BER) and ergodic capacity, etc. ([7,10] and the references therein). However, in the existing models, $\gamma_{e}$ is not directly dealt with but an approximate version $\gamma_{a}=\frac{\gamma_{1}\gamma_{2}}{\gamma_{1}+\gamma_{2}}$ is investigated instead of $\gamma_{e}$ [7,11]. $\gamma_{a}$ is sometimes further approximated by $\gamma_{m}=\min\left\{\gamma_{1},\gamma_{2}\right\}$ [10]. Obviously, $\gamma_{e}<\gamma_{a}\leq \gamma_{m}$ and hence the existing models certainly overestimate the mixed RF/FSO AF relaying performances. In particular, $\gamma_{a}$ can be obtained by taking σ=0 and the resulting amplifying gain can be infinitely large depending on the channel magnitude, which is often referred to an *ideal gain* or a channel-inversion gain [8]. Thus, the performance evaluated with the approximate SNRs usually ignores the *noise propagation* effect in an AF relay system.

The noise propagation is however practically caused by the limited output power at the relay node and becomes especially significant when the first hop SNR is poor or the communication link consists of multiple hops [8,12]. In wireless sensor networks, a battery-charged sensor usually has a very tight budget on transmit power and the first hop SNR at the relay becomes inevitably low. Thus, mixed RF/FSO AF relaying application in wireless sensor networks should be adequately evaluated by including the noise propagation effect.

In this paper, we provide an exact analysis on the probability distribution of $\gamma_{e}$ and illustrate the noise propagation effect on the performances. Unlike the existing methodology based on a moment generating function (MGF) technique (e.g., [7,10]), the analysis in this paper mainly relies on the binomial-equation theorem [13] and the power series expansion of an exponential function [14]. Counting the noise power amplified at the relay, we could see the performance degradation due to the noise propagation in comparison to the approximate results. We assume that RF and FSO link suffer from Nakagami-*m* and Gamma-Gamma fading with pointing errors, respectively. In [7] and [10], more generalized settings are used for modeling the fading channels but, to the best of our knowledge, no existing works have provided the exact statistical analysis of the mixed RF-FSO AF system with the power-constrained variable amplifying gain. The analysis provided in this paper is verified by simulation. Numerical investigation on the performance degradation shows that if the SNR (of each hop) is 10 dB, compared with the performances approximated by adopting $\gamma_{a}$, the exact OP is degraded by up to 18.5%, the average BER by up to 12.17% and the ergodic capacity by up to 8.08%. Though the performance gap diminishes to around 1% if the SNR is greater than 30 dB, the exact analysis seems greatly important in the medium- or the low-SNR ranges.

## 2. System Model and Fading Statistics

**Figure 1:**
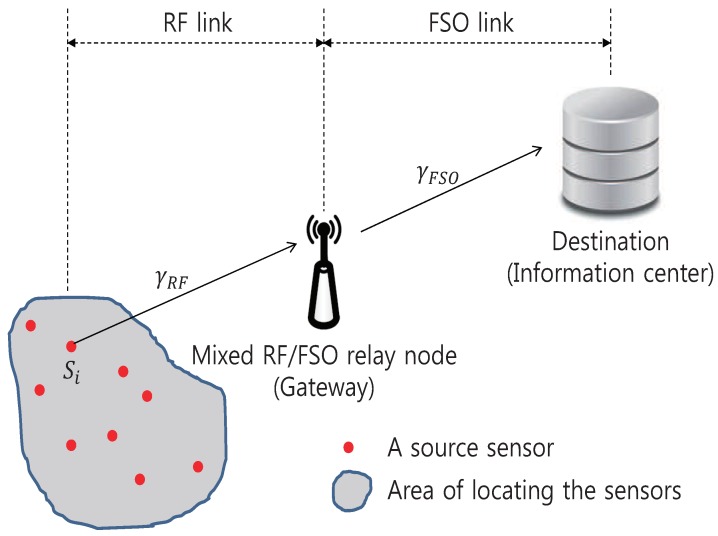
The system model.

We consider an asymmetric dual-hop mixed RF/FSO AF relaying communication system where a source sensor (S) and a destination data center (D) are communicating through an intermediate relay node (R) (as in Figure 1). We assume that S-R link is RF and R-D link is FSO (FSO/RF link also can be similarly treated with the following analysis). This model is applicable to any sensor-network scenario that consists of low-power RF link among remote sensors (or terminals) and mid/long-distance FSO-relaying link between an intermediate cluster head (or gateway) and an information sink. In *Project Loon*, for an example, hot air balloons are connected using FSO mesh links to provide LTE-level internet services to infrastructure-poor areas [15]. Airborne weather sensors linked by RF in a balloon also can be connected to an information center on the ground using FSO link.

Let $\gamma_{RF}$ and $\gamma_{FSO}$ denote the SNR of RF and FSO link, respectively. Since we consider a tightly power-constrained amplifying gain at the relay [8,9], the end-to-end SNR is given by

(3) $$\begin{array}{c} \gamma_{e}=\frac{\gamma_{RF}\gamma_{FSO}}{\gamma_{RF}+\gamma_{FSO}+1}. \end{array}$$

It is noted that the orders of RF-FSO and FSO-RF are equivalent in terms of the end-to-end SNR if the tightly power-constrained amplifying gain is used.

If we assume that RF link suffers from Nakagami-*m* fading, $\gamma_{RF}$ follows a Gamma distribution with fading parameter *m* and Ω and an average SNR ${\bar{\gamma }}_{RF}$, the probability density function (PDF) of which is given by

(4) $$\begin{array}{c} f_{\gamma_{RF}}\left(x\right)={\left(\frac{m}{\Omega {\bar{\gamma }}_{RF}}\right)}^{m}\frac{x^{m-1}}{\Gamma (m)}e^{-\frac{m}{\Omega {\bar{\gamma }}_{RF}}x}, \end{array}$$

where Γ(·) is a Gamma function such that $\Gamma \left(x\right)=\int_{0}^{\infty }z^{x-1}e^{-z}dz$. For FSO link, let η and *I* denote the effective photo-electric conversion ratio and the channel coefficient of the FSO link, respectively. Then the SNR of FSO link is modeled by $\gamma_{FSO}={\left(\eta I\right)}^{w}/N_{0}$ [7], where $N_{0}$ is the variance of zero-mean white Gaussian noise at the destination and w∈{1,2} represents a specific detection technique (i.e., w=1 and 2 account for HD and IM/DD, respectively). It is further assumed that $I=I_{l}I_{p}I_{f}$, where $I_{l}$ is the path loss, $I_{p}$ the pointing error, and $I_{f}$ is the fading caused by atmospheric turbulence. We assume $I_{l}=1$, since $I_{l}$ is deterministic [16], whereas $I_{p}$ and $I_{f}$ are probabilistic with PDFs $f_{I_{p}}\left(x\right)$ and $f_{I_{f}}\left(x\right)$. According to [17], the PDF of $I_{p}$ is given by

(5) $$\begin{array}{c} f_{I_{p}}\left(x\right)=\frac{\delta^{2}}{\Phi^{\delta^{2}}}x^{\delta^{2}-1},\;\;0\leq x\leq \Phi , \end{array}$$

where δ is a ratio between the equivalent beam radius and the pointing error displacement standard deviation at the receiver (i.e., the larger the delta, the smaller the effect of the pointing error) and Φ is a fraction of the collected power at radial displacement r=0. The PDF of $I_{f}$ is given in [3] such that

(6) $$\begin{array}{c} f_{I_{f}}\left(x\right)=\frac{2{\left(\alpha \beta \right)}^{(\alpha +\beta )/2}}{\Gamma (\alpha )\Gamma (\beta )}x^{(\alpha +\beta )/2-1}K_{\alpha -\beta }\left(2\sqrt{\alpha \beta x}\right),\;\;x>0, \end{array}$$

where α and β are atmospheric turbulence parameters and $K_{a}\left(\cdot \right)$ is the modified Bessel function of the second kind of order *a*. Using the generalized power series representation of the modified Bessel function of the second kind [18] (8.445, 8.485), the PDF in (6) can be expressed as

(7) $$\begin{array}{c} f_{I_{f}}\left(x\right)=\sum_{j=0}^{\infty }a_{j}\left(\alpha ,\beta \right)x^{j+\beta -1}+a_{j}\left(\beta ,\alpha \right)x^{j+\alpha -1}, \end{array}$$

where

(8) $$\begin{array}{c} a_{j}\left(\alpha ,\beta \right)=\frac{\pi {\left(\alpha \beta \right)}^{j+\beta }\csc\left(\pi \left(\alpha -\beta \right)\right)}{\Gamma (\alpha )\Gamma (\beta )\Gamma (j-\alpha +\beta +1)\Gamma (j+1)}, \end{array}$$

and furthermore α−β should not be an integer [18] (8.485). Using (7), the cumulative distribution function (CDF) of $I_{f}$ can be derived as

(9) $$\begin{array}{c} F_{I_{f}}\left(x\right)=\sum_{j=0}^{\infty }\frac{a_{j}\left(\alpha ,\beta \right)}{j+\beta }x^{j+\beta }+\frac{a_{j}\left(\beta ,\alpha \right)}{j+\alpha }x^{j+\alpha }. \end{array}$$

Using (5) and (9), the CDF of $I=I_{l}I_{p}I_{f}$ can be expressed by

(10) $$\begin{array}{ll} F_{I}\left(x\right) & =\int_{0}^{\Phi }F_{I_{f}}\left(x/t\right)f_{I_{p}}\left(t\right)dt\\ & =\sum_{j=0}^{\infty }b_{j}\left(\alpha ,\beta ,\delta \right)x^{j+\beta }+b_{j}\left(\beta ,\alpha ,\delta \right)x^{j+\alpha }, \end{array}$$

where $b_{j}\left(\alpha ,\beta ,\delta \right)=a_{j}\left(\alpha ,\beta \right)\delta^{2}/\left\{\left(j+\beta \right)\left(\delta^{2}-j-\beta \right)\Phi^{j+\beta }\right\}$. By using (10), the CDF of $\gamma_{FSO}$ is finally obtained by

(11) $$\begin{array}{c} F_{\gamma_{FSO}}\left(x\right)=\sum_{j=0}^{\infty }\frac{b_{j}\left(\alpha ,\beta ,\delta \right)}{{\left(\eta^{w}\mu_{w}\right)}^{\frac{j+\beta }{w}}}x^{\frac{j+\beta }{w}}+\frac{b_{j}\left(\beta ,\alpha ,\delta \right)}{{\left(\eta^{w}\mu_{w}\right)}^{\frac{j+\alpha }{w}}}x^{\frac{j+\alpha }{w}}, \end{array}$$

where $\mu_{w}$ denotes the average electrical SNR. More specifically for $\mu_{w}$, when w=1, $\mu_{1}={\bar{\gamma }}_{FSO}$ and when w=2, $\mu_{2}={\bar{\gamma }}_{FSO}\alpha \beta \delta^{2}\left(\delta^{2}+2\right)/\left\{\left(\alpha +1\right)\left(\beta +1\right){\left(\delta^{2}+1\right)}^{2}\right\}$ [5].

## 3. Exact Statistical Analysis of $\gamma_{e}$

By introducing exclusive events that construct a partition: $\left\{\gamma_{RF}\geq x\right\}$ and $\left\{\gamma_{RF}<x\right\}$, the CDF of $\gamma_{e}$ is equivalently expressed by

(12) $$\begin{array}{ll} F_{\gamma_{e}}\left(x\right) & =\mathrm{Pr}\left\{\frac{\gamma_{RF}\gamma_{FSO}}{\gamma_{RF}+\gamma_{FSO}+1}<x\right\}\\ = & \mathrm{Pr}\left\{\gamma_{FSO}<\frac{x(\gamma_{RF}+1)}{\gamma_{RF}-x},\gamma_{RF}\geq x\right\}+\mathrm{Pr}\left\{\gamma_{RF}<x\right\}\\ = & \underset{F_{\gamma ,1}}{\underset{︸}{\int_{0}^{x}f_{\gamma_{RF}}\left(t\right)dt}}+\underset{F_{\gamma ,2}}{\underset{︸}{\int_{x}^{\infty }F_{\gamma_{FSO}}\left(\frac{x(t+1)}{t-x}\right)f_{\gamma_{RF}}\left(t\right)dt}}. \end{array}$$

$F_{\gamma ,1}$ is the CDF of $\gamma_{RF}$ given by [18] (3.381.1)

(13) $$\begin{array}{c} F_{\gamma ,1}=\frac{\gamma (m,\frac{mx}{\Omega {\bar{\gamma }}_{RF}})}{\Gamma (m)}, \end{array}$$

where γ(·,·) is a lower incomplete gamma function defined by $\gamma \left(s,x\right)=\int_{0}^{x}t^{s-1}e^{-t}dt$.

(14) $$F_{\gamma ,2}=\int_{x}^{\infty }\left[\sum_{j=0}^{\infty }\frac{b_{j}\left(\alpha ,\beta ,\delta \right)}{{\left(\frac{\eta^{w}\mu_{w}}{x}\right)}^{\frac{j+\beta }{w}}}{\left(\frac{t+1}{t-x}\right)}^{\frac{j+\beta }{w}}+\frac{b_{j}\left(\beta ,\alpha ,\delta \right)}{{\left(\frac{\eta^{w}\mu_{w}}{x}\right)}^{\frac{j+\alpha }{w}}}{\left(\frac{t+1}{t-x}\right)}^{\frac{j+\alpha }{w}}\right]{\left(\frac{m}{\Omega {\bar{\gamma }}_{RF}}\right)}^{m}\frac{t^{m-1}}{\Gamma (m)}e^{-\frac{mt}{\Omega {\bar{\gamma }}_{RF}}}dt$$

(15) =(a)∑j=0∞∑ℓ=0∞∑k=0∞(j+βwℓ)(−j+βwk)bj(α,β,δ)(ηwμw/x)j+βw+(j+αwℓ)(−j+αwk)bj(β,α,δ)(ηwμw/x)j+αw×(−x)kΓ(m)(mΩγ¯RF)m∫x∞tm−ℓ−k−1e−mtΩγ¯RFdt,x≥1∑j=0∞∑ℓ=0∞∑k=0∞(j+βwℓ)(−j+βwk)bj(α,β,δ)(−x)kΓ(m)(ηwμw/x)j+βw(mΩγ¯RF)m×(∫1∞tm−ℓ−k−1e−mtΩγ¯RFdt+∫x1tm+ℓ−k−1−j+βwe−mtΩγ¯RFdt)+(j+αwℓ)(−j+αwk)bj(β,α,δ)(−x)kΓ(m)(ηwμw/x)j+αw(mΩγ¯RF)m×(∫1∞tm−ℓ−k−1e−mtΩγ¯RFdt+∫x1tm+ℓ−k−1−j+αwe−mtΩγ¯RFdt),x<1

(16) Fγe(x)=∑j=0∞∑ℓ=0∞∑k=0∞(j+βwℓ)(−j+βwk)bj(α,β,δ)(ηwμw/x)(j+β)/w+(j+αwℓ)(−j+αwk)bj(β,α,δ)(ηwμw/x)(j+α)/w×(−x)kΓ(m)(mΩγ¯RF)ℓ+kΓ(m−ℓ−k,mxΩγ¯RF)+γ(m,mxΩγ¯RF)Γ(m),x≥1∑j=0∞∑ℓ=0∞∑k=0∞(−x)kΓ(m)[(j+βwℓ)(−j+βwk)bj(α,β,δ)(ηwμw/x)(j+β)/w{(mΩγ¯RF)ℓ+kΓ(m−ℓ−k,mΩγ¯RF)+∑u=0∞(−1)uΓ(u+1)(mΩγ¯RF)m+u1−xm+ℓ−k+u−(j+β)/wm+ℓ−k+u−(j+β)/w}+(j+αwℓ)(−j+αwk)bj(β,α,δ)(ηwμw/x)(j+α)/w{(mΩγ¯RF)ℓ+k×Γ(m−ℓ−k,mΩγ¯RF)+∑u=0∞(−1)uΓ(u+1)(mΩγ¯RF)m+u1−xm+ℓ−k+u−(j+α)/wm+ℓ−k+u−(j+α)/w}]+γ(m,mxΩγ¯RF)Γ(m),x<1

And furthermore $F_{\gamma ,2}$ is given in (15) at the top of this page. The equality $\overset{\left(a\right)}{=}$ in (15) is obtained by applying Newton’s generalized binomial theorem [13] to ${\left(\frac{t+1}{t-x}\right)}^{\frac{j+\alpha (\mathrm{or}\;\beta )}{w}}$ in (14). And by applying [18] (3.381.3) to $\int_{x(\mathrm{or}\;1)}^{\infty }t^{m-\ell -k-1}e^{-\frac{mt}{\Omega {\bar{\gamma }}_{RF}}}dt$ in (15) and by applying the power series (especially with Maclaurin series) expansion to the exponential function in $\int_{x}^{1}t^{m+\ell -k-1-\frac{j+\alpha (\mathrm{or}\;\beta )}{w}}e^{-\frac{mt}{\Omega {\bar{\gamma }}_{RF}}}dt$ in (15), the CDF of $\gamma_{e}$ is finally can be obtained by (16).

## 4. Application of $F_{\gamma_{e}}$ for Performance Evaluation

Using $F_{\gamma_{e}}$ obtained in (16), the performance evaluation could be done as follows.

### 4.1. Outage Probability

The outage probability is defined by the probability that the instantaneous output SNR $\gamma_{e}$ falls below a predetermined threshold $\gamma_{th}$. Since we already have the CDF of $\gamma_{e}$, the outage probability is simply given by

(17) $$\begin{array}{c} P_{out}=\mathrm{Pr}\left\{\gamma_{e}<\gamma_{th}\right\}=F_{\gamma_{e}}\left(\gamma_{th}\right). \end{array}$$

### 4.2. Average BER

The average BER for a variety of binary modulations is given by

(18) $$\begin{array}{c} P_{b}=\frac{q^{p}}{2\Gamma (p)}\int_{0}^{\infty }e^{-qx}x^{p-1}F_{\gamma_{e}}\left(x\right)dx, \end{array}$$

where *p* and *q* are parameters that change for different modulation schemes [19].

### 4.3. Ergodic Capacity

The ergodic capacity is defined by $\bar{C}=E\left[\log_{2}\left(1+c\gamma_{e}\right)\right]$ as in [7,10], where c=1 for HD and c=e/(2π) for IM/DD. By employing a part-by-part integration method, we can write it in terms of the $F_{\gamma_{e}}\left(x\right)$ as

(19) $$\begin{array}{c} \bar{C}=\frac{c}{\ln(2)}\int_{0}^{\infty }\frac{1-F_{\gamma_{e}}\left(x\right)}{1+cx}dx. \end{array}$$

## 5. Numerical Results

In the simulation, the Gamma-Gamma fading channel for FSO link is assume to follow weak (α=2.902 and β=2.51), moderate (α=2.296 and β=1.822), and strong (α=2.064 and β=1.342) turbulent FSO channel conditions [20]. We also assume that η=0.8 and Φ=1 at r=0 for FSO link. For the Nakagami-*m* faded RF link, m=1.1 and Ω=1 are assumed. The average SNRs for the FSO and the RF links are assumed to be the same and denoted by $\bar{\gamma }={\bar{\gamma }}_{FSO}={\bar{\gamma }}_{RF}$. For the outage threshold, $\gamma_{th}=1$ is assumed. When numerically evaluating the infinite series contained in the exact CDF obtained in (16), we use an upper bound on sequence indices j,l,k such that j,l,k≤50.

**Figure 2:**
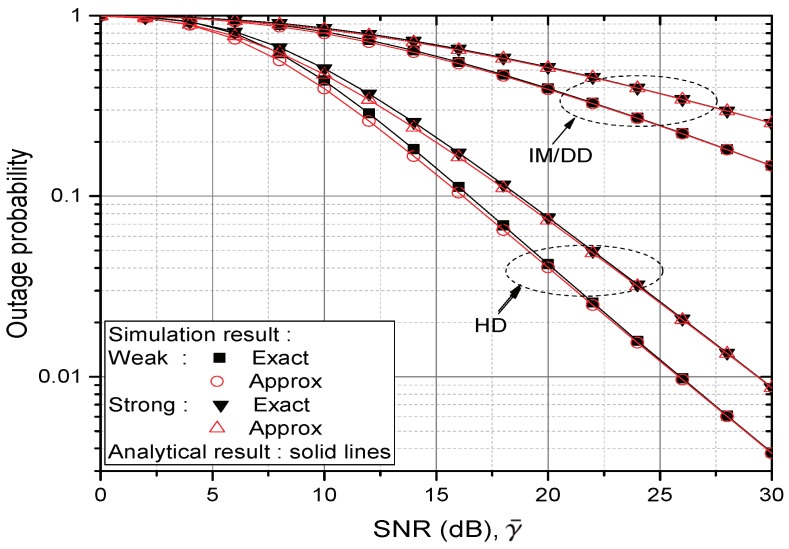
Comparison of CDFs (equivalently, OP) from $\gamma_{e}$ and $\gamma_{a}$; δ=1 under weak or strong turbulence, respectively.

**Figure 3:**
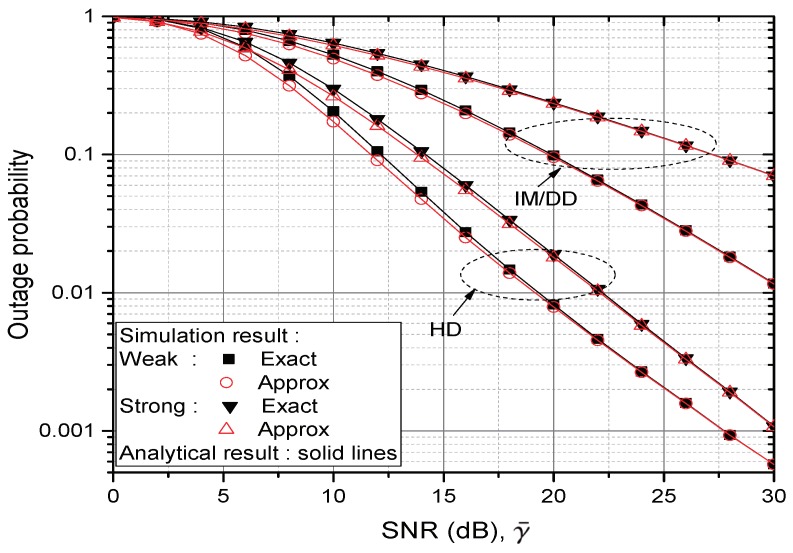
Comparison of CDFs (equivalently, OP) from $\gamma_{e}$ and $\gamma_{a}$; δ=6.7 under weak or strong turbulence, respectively.

Figure 2 and Figure 3 verify the analytical result on the CDF of $\gamma_{e}$ (equivalently, OP performance) by comparing it with the simulation result. Different pointing error assumptions δ=1 and 6.7 are used in Figure 2 and Figure 3, respectively. In both of the Figures, it is seen that the analytical result is exactly matched with the simulation. In the Figures, the gap between “Exact” and “Approx” result reveals the effect of noise amplification. It becomes large when the test environment changes from high to low SNR, from IM/DD to HD, from strong to weak turbulence and from high to low pointing error assumption, respectively. At SNR 10 dB, the degradation in OP due to the noise propagation is about 10.71% at HD receiver in weak turbulence environments in Figure 2. It diminishes to 7.98% in strong turbulence but increases to 18.5% in Figure 3 where lower pointing error is assumed. If IM/DD receiver is considered, the above three percentages reduce to 2.43%, 1.69% and 6.58%, respectively. If the SNR is greater than 30 dB, the gap in every tested point becomes less than 1%.

In Table 1, the performance degradation percentage is shown for the average BER (letting p=q=1) and the ergodic capacity ($\bar{C}$). The trend in changing the degradation volume for the average BER is similar to the result for OP. However, for the ergodic capacity, the trend seems different (actually the opposite) according to the detection type, the pointing error assumption, and the turbulent parameter especially if HD detection is assumed. For any case, the gap is from 2.44% to 12.17% at 10-dB SNR but it diminishes to less than 1% at 30-dB SNR.

## 6. Conclusions

In this paper, an exact CDF expression on end-to-end SNR is provided for a dual-hop mixed RF/FSO AF system, which can be used to count the noise propagation effect in performance evaluation of OP, average BER and ergodic capacity. The numerical result reveals that the performance degradation due to the inclusion of the noise propagation is about 2–12% at 10-dB SNR depending on the transmission environments. Though the performance gap reduces below 1% if the SNR is greater than 30 dB, adequate evaluation on the noise propagation effect seems still important especially when an energy-limited or a multihop RF/FSO AF system is considered, which could be a future research topic.

## Figures and Tables

**Table 1:** Performance degradation percentage due to the noise propagation effect in comparison with the approximate results from $\gamma_{a}$.

		HD				IM/DD			
		δ = 1		δ = 6.7		δ = 1		δ = 6.7	
Turbulence	SNR	BER	$\bar{C}$	BER	$\bar{C}$	BER	$\bar{C}$	BER	$\bar{C}$
Weak	10	9.28	5.69	12.17	3.97	3.35	8.08	6.88	5.64
	20	3.72	0.46	3.72	0.27	1.30	0.90	2.79	0.55
	30	0.52	0.03	0.38	0.02	0.25	0.08	0.54	0.04
Moderate	10	8.79	5.74	11.61	4.06	2.83	7.97	5.57	5.70
	20	3.59	0.47	4.12	0.29	1.09	0.92	2.08	0.58
	30	0.42	0.03	0.50	0.02	0.21	0.08	0.45	0.05
Strong	10	7.76	5.83	10.26	4.25	2.44	7.82	4.64	5.71
	20	3.09	0.50	4.13	0.32	0.92	0.92	1.64	0.61
	30	0.47	0.04	0.74	0.02	0.18	0.09	0.29	0.05

## Abbreviations

The following abbreviations are used in this manuscript:

AF	Amplifying-and-forwarding
BER	Bit-error rate
CDF	Cumulative distribution function
DF	Decoding-and-forward
DGG	Double generalized Gamma
EGK	Extended generalized-*K*
FSO	Free-space optical
HD	Heterodyne detection
IM/DD	Intensity-modulation/direct-detection
MGF	Moment generating function
OP	Outage probability
PDF	Probability density function
RF	Radio frequency
SNR	Signal-to-noise power ratio
